# Gene expression and hepatitis C virus infection

**DOI:** 10.1136/gut.2008.166348

**Published:** 2008-12-10

**Authors:** T Asselah, I Bièche, A Sabbagh, P Bedossa, R Moreau, D Valla, M Vidaud, P Marcellin

**Affiliations:** 1 INSERM, U773, Centre de Recherche Bichat-Beaujon CRB3, Paris, France; 2 Université Denis Diderot-Paris 7, Site Bichat, France; 3 Service d’hépatologie, Hôpital Beaujon, Clichy, France; 4 INSERM, U745, Université René Descartes, Paris, France; 5 Service de Génétique Moléculaire, Hôpital Beaujon, Clichy, France; 6 Service d’Anatomie-Pathologie, Hôpital Beaujon, Clichy, France

## Abstract

Hepatitis C virus (HCV) is a major cause of chronic liver disease, with about 170 million people infected worldwide. Up to 70% of patients will have persistent infection after inoculation, making this disease a significant cause of morbidity and mortality.

The severity of disease varies widely, from asymptomatic chronic infection to cirrhosis and hepatocellular carcinoma. Since the discovery of HCV, the treatment of hepatitis C has considerably improved. Recently, combination of pegylated interferons with ribavirin gives a response rate of about 55%. Treatment is indicated in patients with moderate or severe fibrosis. The tolerability of combination treatment is relatively poor, with a frequent flu-like syndrome and an impaired quality of life.

In addition to viral and environmental behavioural factors, host genetic diversity is believed to contribute to the spectrum of clinical outcomes in HCV infection. The sequencing of the human genome, together with the development of high-throughput technologies that measure the function of the genome, have afforded unique opportunities to develop profiles that can distinguish, identify and classify discrete subsets of disease, predict the disease outcome or predict the response to treatment. This paper reviews the published literature on gene expression associated with HCV infection (HCV infection, fibrosis progression), and also according to response to treatment.

## PATHOPHYSIOLOGY

Hepatitis C virus (HCV) is a major cause of chronic liver disease, with ∼170 million people infected worldwide^1^ (box 1). HCV belongs to the Flaviviridae family (hepacivirus genus), and is an enveloped virus with a 9.6 kb single-stranded RNA genome^2^^–^7 (box 2). Until recently, the absence of a cell culture model supporting full replication of HCV and of convenient animal models has limited knowledge of the HCV life cycle and the testing for antiviral molecules. The chimpanzee is the only animal model for HCV infection.^8^ The development of a subgenomic HCV RNA replicon capable of replication in the human hepatoma cell line, Huh 7, was a significant advance.^9^ ^10^ Recently, complete replication of HCV in cell culture has been achieved.^11^ ^12^ These models will improve our understanding of HCV replication and the testing for antiviral molecules. HCV infection is associated with a spectrum of extrahepatic manifestations, mainly mixed cryoglobulin.^13^

## INTERFERON SIGNALLING AND HCV INFECTION

**Figure 1:**
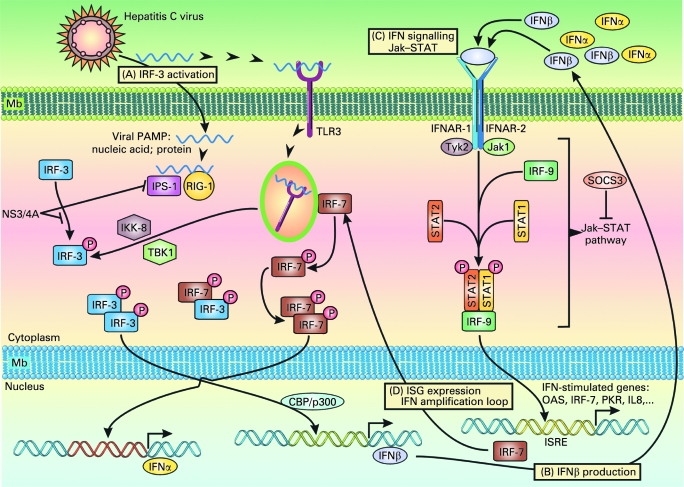
Hepatitis C virus (HCV) infection and immune response. HCV can induce several signalling pathways. (A) Toll-like receptor (TLR) signalling. Activation of TLR3 leads to the recruitment of IκB kinase (IKK)-related kinases, TANK-binding kinase 1 (TBK1, also known as T2K and NAK) and IKKi (also known as IKKϵ). These kinases, together with adaptors TANK and NAP1, catalyse the phosphorylation of interferon (IFN) regulatory factor-3 (IRF-3). TLR3 engagement also results in the activation of transcription factors AP-1 and nuclear factor-κB (NF-κB). Phosphorylated IRF-3 forms a dimer, translocates into the nuclei, binds to DNA and regulates the expression of IFNβ in collaboration with AP-1 and NF-κB. The HCV NS3-4A serine protease may block the phosphorylation and effector action of IRF-3. (B) Retinoic acid-inducible gene-I (RIG-I)-like RNA helicase signalling. After recognition of viral RNA, RIG-I and also Mda5 (not shown) recruit IFNβ promoter stimulator-1 (IPS-1, also known as MAVS, Cardif and VISA) via CARD–CARD (caspase recruitment domain) interaction. IPS-1 is localised to mitochondria and acts as an adaptor that plays a critical role in the activation of IRF-3 and IRF-7 in a TBK1- and IKKi-dependent manner. IPS-1 also interacts with the Fas-associated death domain protein (FADD), which is required for the activation of IRF-3 and NF-κB. IRF-7 forms a dimer, translocates into the nucleus to induce IFNα/β; homodimers of IRF-3 collaborate with NF-κB to induce IFNβ. IPS-1 is targeted and inactivated by NS3-4A, a serine protease from HCV known to block IFNβ production. (C) IFN signalling. Endogenous IFNα/β bind to a common receptor expressed at the surface of target cells. Receptor engagement leads to the activation of STAT1 (signal transducer and activator of transcription 1) and STAT2, which, together with ISGF3G (IFN-stimulated gene factor 3, γ subunit)/IRF-9, bind to *cis*-acting IFN-stimulated response elements (ISREs) (D), thereby activating the transcription of IFNα/β-inducible genes such as those encoding RNase L and protein kinase R (PKR) which degrade viral RNAs and block their translation. Also, the HCV core protein has been shown to induce the expression of SOCS3 (suppressor of cytokine signalling 3), which can suppress Jak (Janus kinase)–STAT signalling events and block the IFN-induced formation of ISGF3.^58^ PAMP, pathogen-associated molecular pattern.

Endogenous type I interferons (IFNs) are the main antiviral cytokines. HCV infection may activate host signalling pathways that induce type I IFNs.^14^^–^16 It should be remembered that the double-stranded (ds) RNA virus induces the host immune response; dsRNA which is a pathogen-associated molecular pattern, is recognised by pattern recognition receptors (PRRs) such as Toll-like receptor 3 (TLR3) and retinoic acid-inducible gene-I (RIG-I) (fig 1). Although HCV is a single-stranded RNA, the fact that replication of the HCV genome is catalysed by its RNA-dependent RNA polymerase, NS5B, suggests that dsRNA may be formed during the HCV life cycle and activate PRRs. Activation of TLR3 via the adaptor TRIF leads to phosphorylation of IFN regulatory factor-3 (IRF-3) and activation of transcription factors AP-1 and nuclear factor-κB (NF-κB). Phosphorylated IRF-3 forms a dimer, translocates into the nuclei, binds to DNA and regulates the expression of IFNβ expression in collaboration with AP-1 and NF-κB. After recognition of viral RNA, RIG-I and Mda5 recruit IFNβ promoter stimulator-1 (IPS-1, also known as Cardif).^15^ IPS-1 is localised to mitochondria and plays a critical role in the activation of IRF-7, IRF-3 and NF-κB. IRF-7 forms a dimer, translocates into the nucleus to induce IFNα/β; homodimers of IRF-3 collaborate with NF-κB to induce IFNβ. IFNα/β of autocrine/paracrine sources bind to a common receptor expressed at the cell surface. Receptor engagement causes the activation of Jak (Janus kinase)–STAT (signal transducers and activators of transcription) signalling which, together with ISGF3G (IFN-stimulated gene factor 3, γ subunit)/IRF-9, binds to IFN-stimulated response elements (ISREs), thereby activating the transcription of IFNα/β-inducible genes.^14^ This results in the production of effector molecules, such as RNase L and protein kinase R (PKR), that will degrade viral RNAs and block their translation.

It should be noted that HCV RNA encodes specific proteins that may inhibit the induction of type I IFNs. An example is the NS3-4A protease of HCV, which blocks dsRNA-induced IFN production by interfering with IRF-3 phosphorylation.^16^ NS3-4A cleaves the C-terminal region of IPS-1/Cardif, causing disruption of NF-κB and IRF-3 activation, probably due to mislocalisation of cleaved IPS-1/Cardif from mitochondria. NS3-4A also mediates TRIF proteolysis, suggesting multiple functions for this protease. Thus, HCV proteins may block both the TLR- and RIG-I–Mda5-dependent signalling pathways to antagonise type I IFN induction. Thus, the NS3-4A protease is a dual therapeutic target, whose inhibition may block viral replication and restore IRF-3 control of HCV infection.

HCV-related effects may also attenuate IFN signalling. Proteins called suppressor of cytokine signalling (SOCS) are known to inhibit cytokine signalling via Jak–STAT. The HCV core protein has been shown to induce the expression of SOCS3, which can suppress Jak–STAT signalling events and block the IFN-induced formation of ISGF3.^17^ HCV protein expression in liver cells is associated with induction of the protein inhibitor of activated STAT (PIAS) expression and inhibition of STAT function. Patients with chronic HCV infection have been shown to exhibit high levels of interleukin 8 (IL8) in the liver.^18^ The biological activity of IL8 interferes with IFN signalling events that result in ISGF3 recruitment and function.

**Box 1 Natural history of hepatitis C virus infection**

The natural history of hepatitis C virus infection is influenced by both genetic and environmental factors. T lymphocytes, which are important in terms of both viral clearance and hepatotoxicity, are stimulated in their response after presentation of foreign material by antigen-presenting cells in association with major histocompatibility complex (MHC) molecules. Several studies, predominantly in Caucasians, have found associations of the human leucocyte antigen (HLA) class II alleles DQB1 0301 and DRB111 with self-limiting infection populations. Also a high quasispecies population is believed to be associated with chronicity.

The major factors known to be associated with fibrosis progression are male gender, older age at infection and excessive alcohol consumption. Interestingly, viral factors such as viral load and genotype do not seem to influence the progression rate significantly. Progression of fibrosis is more rapid in immunocompromised patients. Insulin resistance may also contribute to more rapid progression of fibrosis.^31^^–^33

**Figure:**
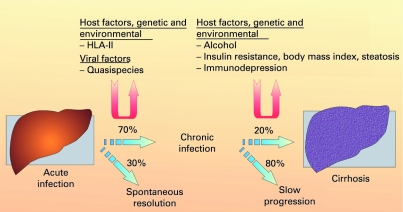

**Box 2 HCV cycle**

HCV is a member of the Flaviviridae family, genus Hepacivirus. Error-prone replication of HCV, resulting in a complex quasispecies population within each infected individual, enables rapid adaptation to changing environments. Six HCV genotypes and a large number of subtypes have been identified.^3^ The HCV virion is made of a single-stranded positive RNA genome, contained in a capsid, itself enveloped by a lipid bilayer within which two different glycoproteins are anchored. The HCV life cycle starts with virion attachment to its specific receptor. Several candidate molecules have been suggested to play a role in the receptor complex, including tetraspanin CD81, the scavenger receptor BI (SR-BI), the adhesion molecules DC-SIGN and L-SIGN, and the low-density lipoprotein (LDL) receptor.^4^ Recently, the tight junction components claudins (mainly CLDN-1, CLDN-6 and CLDN-9) have been identified as additional key factors for HCV infection.^5^ ^6^ The CD81 partner EWI-2wint inhibits HCV entry, suggesting that, in addition to the presence of specific entry factors in the hepatocytes, lack of a specific inhibitor can contribute to the hepatotropism of HCV.^7^ The HCV RNA genome serves as a template for viral replication and as a viral mRNA for viral production. It is translated into a polyprotein which is cleaved by proteases.^8^^–^10 All the HCV enzymes—NS2-3 and NS3-4A proteases, NS3 helicase and NS5B RdRp—are essential for HCV replication, and are therefore potential drug discovery targets.

**Figure:**
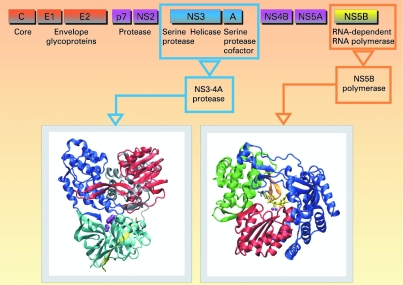

Together, these findings suggest that HCV modulation of IFN induction and signalling limits the expression of IFN-stimulated genes, allowing HCV to evade the antiviral actions of the host response.

## MICRORNA AND HCV INFECTION

**Figure 2:**
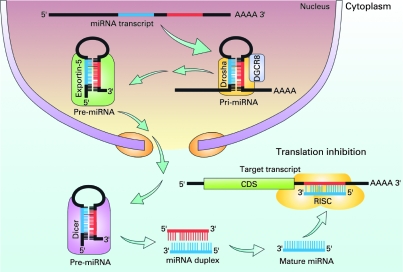
Biogenesis of microRNAs (miRNAs). miRNA is transcribed mainly by RNA polymerase II. The primary transcript (pri-miRNA that can be quite large) is processed in the nucleus by an enzymatic complex that includes the enzymes Drosha and DGCR8, which leads to the formation of a 70–100 nucleotide hairpin precursor named pre-miRNA. This second precursor binds exportin-5 in the nucleus and is transported to the cytoplasm, where it is cleaved by Dicer into mature miRNA. This mature miRNA is incorporated into a ribonucleoprotein complex called the “RNA-induced silencing complex” (RISC), and guides the RISC to the 3′-unstranslated region of the target mRNAs. On the basis of the degree of homology between the miRNA and the mRNA, RISC can inhibit mRNA function by either promoting its degradation or inhibiting its translation. It is believed that each miRNA can target up to 200 mRNAs, and multiple miRNAs can target a given transcript. Therefore, the potential regulatory circuitry afforded by miRNA is extremely complex.

MicroRNAs (miRNAs) are a class of small non-coding RNA molecules of 20–22 nucleotides that control gene expression by targeting mRNAs for translational repression or cleavage.^19^ The biogenesis of miRNAs involves a complex protein system, including RNA polymerase II and the RNase IIIs Drosha and Dicer (fig 2). A total of nearly 700 human miRNAs have been reported so far (the April 2008 release of miRBase Sequence Database-Release 11.0, at the Sanger Institute), but a total number of >1000 human miRNAs is estimated.^20^ miRNAs are a new player among gene regulation mechanisms and, although their functions have not been fully clarified, they include the regulation of development, cell differentiation, proliferation and apoptosis.

Silencing of the RNAase III Dicer has been shown to inhibit HCV replication by ∼7-fold.^21^ Moreover, depletion of Huh7 hepatoma of the liver-specific miRNA miR-122 (that directly interacts with the 5′ end of the HCV RNA genome) was found to be associated with inhibition of HCV replication and infectious viral production.^22^ These findings suggest that HCV takes advantage of the presence of miR-122 in hepatocytes, which may be a target for novel approaches in the treatment of HCV infection. Interestingly, IFNβ was found to modulate the expression of numerous cellular miRNAs rapidly in vitro, and eight of these IFNβ-induced miRNAs were shown to have sequence-predicted targets within the HCV genomic RNA.^23^ Moreover, IFNβ results in a significant reduction in miR-122 expression. These findings strongly support the notion that the human organism uses cellular miRNAs to combat HCV infection through the IFN system, and also adds a new component to the antiviral arsenal of IFNs, which are the most common treatment of HCV infection.

## HCV IN HUMAN LIVER SAMPLES: CONTRIBUTION FROM GENE EXPRESSION STUDIES

The recent development of efficient tools for large-scale analysis of gene expression has provided new insights into the role of gene networks and regulatory pathways in various tumour processes.^24^ These tools include microarrays, which can analyse the expression of thousands of genes at one time, and real-time reverse transcription-PCR (RT-PCR) assays for more accurate and quantitative expression analysis of smaller numbers of candidate genes.^18^

Little is known about the molecular mechanisms associated with HCV infection in humans. Liver gene expression was studied, by large-scale real-time RT-PCR, in patients with untreated chronic hepatitis C and mild fibrosis compared with histologically normal controls.^25^ The most significant changes in gene expression mainly affected the transcriptional network regulated by IFNs, including both IFNα/β-inducible genes (*STAT1*, *STAT2*, *ISGF3G/IRF9*, *IFI27*, *G1P3*, *G1P2*, *OAS2* and *MX1*) and IFNγ-inducible genes (*CXCL9*, *CXCL10* and *CXCL11*). Dysregulation of these genes was mainly HCV specific (no upregulation in hepatitis B virus (HBV) infection).

Interestingly, similar results were found in a recent DNA microarray analysis in nine individuals with chronic hepatitis C compared with non-diseased liver controls.^26^ A significant proportion of upregulated genes in chronic hepatitis C were potential ISGs, suggesting an ongoing response to endogenous IFN and/or dsRNA. Viperin, an evolutionarily conserved ISG with antiviral activity against human cytomegalovirus, was significantly elevated in all patients. When Huh7 and HepG2 cells were stimulation with IFNα or IFNγ, viperin was shown to be a predominantly a type I ISG. Furthermore, ISG viperin had anti-HCV activity in vitro.

## THE PROGRESSION OF FIBROSIS IN HEPATITIS C AND EXISTING PREDICTIVE FACTORS

**Figure 3:**
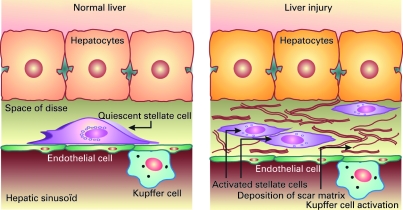
The stellate cell: a key cell implicated in fibrogenesis. Hepatic stellate cells (HSCs) exist in the space between parenchymal cells and sinusoidal endothelial cells of the hepatic lobule, and store vitamin A as retinyl palmitate in lipid droplets in the cytoplasm. In physiological conditions, these cells play pivotal roles in the regulation of vitamin A homeostasis; they express specific receptors for retinol-binding protein (RBP), a binding protein specific for retinol, on their cell surface, and take up the complex of retinol and RBP by receptor-mediated endocytosis. In a normal state, HSCs appear as quiescent vitamin A-storing cells. When activated via several stimuli (infection, alcohol, cytokines, etc.) they acquire a proliferative myofibroblast phenotype. In pathological conditions such as chronic hepatitis C, HSCs lose vitamin A and synthesise a large amount of extracellular matrix components including collagen, proteoglycan and adhesive glycoproteins. Kupffer cells, the resident liver macrophages, remove material from the portal circulation. Kupffer cells may act both as effector cells in the destruction of hepatocytes by producing harmful soluble mediators and as antigen-presenting cells during viral infections of the liver. Moreover, they may represent a significant source of chemoattractant molecules for cytotoxic CD8 and regulatory T cells. Their role in fibrosis is well established as they are one of the main sources of transforming growth factor β1 production, which leads to the transformation of HSCs into myofibroblasts.

**Figure 4:**
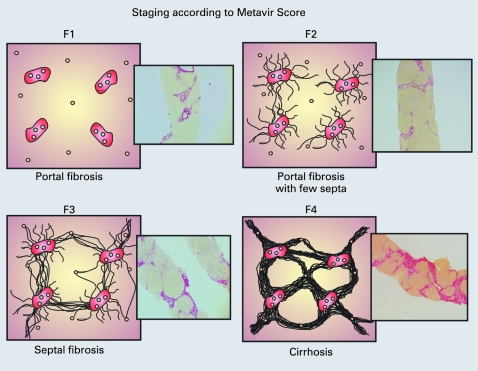
Scoring system for chronic hepatitis C (the Metavir Score System). Liver biopsy remains the gold standard to assess fibrosis. According to the Metavir Score System, fibrosis is scored as F0 (absent), F1 (portal fibrosis), F2 (portal fibrosis with few septa), F3 (septal fibrosis) and F4 (cirrhosis). In addition, necroinflammation activity (A) is graded as A0 (absent), A1 (mild), A2 (moderate) or A3 (severe).

Liver fibrosis is the accumulation in excess of extracellular matrix proteins including collagen.^27^ Fibrogenesis is a complex dynamic process, mediated by necroinflammation and the activation of stellate cells (fig 3). The progression of fibrosis in chronic hepatitis C determines the ultimate prognosis and thus the need for and urgency of treatment.^2829^ Liver biopsy remains the gold standard to assess fibrosis (fig 4). Scoring systems (Knodell, Metavir, Ishak, etc.) provide a semi-quantitative assessment for individual clinical prognosis, cross-sectional and cohort studies, and treatment trials.^30^ The major factors associated with the progression of fibrosis are male gender, older age at infection, excess alcohol consumption and immunosuppression.^31^ ^32^ Insulin resistance may also play a role in the more rapid progression of fibrosis.^33^ The natural history of liver fibrosis in chronic hepatitis C is influenced by both genetic and environmental factors (box 1).

In a recent study, Strnad *et al* analysed genomic DNA from a well-characterised German cohort of 329 patients with chronic hepatitis C and found that previously described and novel keratin 8 (K8) variants are present and collectively associate with the progression of fibrosis.^34^ The unique 100% segregation of the most common K8 variant, R341H, with an intronic deletion suggests that one of these two genetic changes might lead to the other. One potential mechanism which may cause K8 and K18 variants to predispose to liver injury is their established role in hepatocyte cytoprotection, which has been clearly documented in several transgenic mouse studies that express K8/K18 mutants or that lack K8/K18.^35^

## LARGE-SCALE AND GENOME-WIDE ASSOCIATION STUDIES IN THE PROGRESSION OF FIBROSIS

Until recently, almost all association studies performed to identify genetic variants associated with HCV clearance, the progression of fibrosis or the response to antiviral treatment have focused on variations in one or a few candidate genes, chosen on the basis either of their known function or from animal models. Recent advances in high-throughput genotyping technology have now made large-scale association studies a reality, and the number of candidate genes investigated in each study is rapidly increasing.

Genome-wide association studies (GWAS) are now ongoing thanks to the rapidly decreasing cost of genotyping, massively multiplexed genotyping technologies, large-scale single nucleotide polymorphism (SNP) discovery as well as the genotyping efforts of the SNP Consortium and the HapMap project. GWAS use dense maps of genetic markers that cover the human genome to look for allele frequency differences between cases and controls. A significant frequency difference suggests that the corresponding region of the genome contains functional DNA variants that influence the trait of interest. The candidate gene strategy has the advantage of focusing resources on a manageable number of genes and polymorphisms that are likely to be important. The strength of the genome-wide screening is its ability to reveal not only genes that would be expected to play a significant role, but also genes that would not, potentially adding new insight into pathophysiology or pharmacology.

A genome-wide scan with putative functional SNPs was carried out on a group of 433 HCV patients (discovery cohort); those markers associated with advanced fibrosis were validated in a separate group of 483 patients (replication cohort).^36^ This functional genome scan included 24 823 gene-centric SNPs composed of either coding functional SNPs (missense, nonsense, acceptor and donor splice sites) or non-coding putative regulatory SNPs that may effect RNA stability (SNPs located at putative transcription factor-binding sites, or 5′-/3′-untranslated regions). A total of 12 248 genes were directly covered by the scan. Most genes were tested with only one SNP; however, 1418 genes had ⩾4 SNPs. Of the 24 823 SNPs genotyped in the discovery cohort, 1609 SNPs showed evidence of association with advanced fibrosis. Of these, 438 SNPs (27.2%) had odds ratios (ORs) >2 or <0.5. The first 100 consecutive SNPs found to be associated with advanced fibrosis in this cohort were then validated by individual genotyping in the replication cohort to confirm the observed associations. Two of these 100 SNPs were found to be significantly associated with advanced fibrosis in both cohorts, with ORs of similar magnitude and direction. The first one was a missense SNP in the DEAD box polypeptide 5 (*DDX5*) gene which was significantly associated with an increased risk of advanced fibrosis in both the discovery and replication cohorts (OR, 1.8 and 2.2, respectively). The second one was a missense SNP in the carnitine palmitoyltransferase 1A (*CPT1A*) gene associated with a decreased risk of advanced fibrosis in both the discovery and replication cohorts (OR, 0.3 and 0.6, respectively). These two novel markers could play a useful role in predicting risk of fibrosis in HCV patients. After the initial findings of two replicated SNPs, the confirmation of all significant SNPs from their genomic scan was completed and 361 SNPs were selected for signature building.^37^ A machine learning tool developed a predictive signature for cirrhosis in Caucasian patients. A seven gene signature that accurately differentiated high risk versus low risk for cirrhosis was developed using WEKA, an open source Machine Learning Workbench.^38^

## CONTRIBUTION OF GENE EXPRESSION STUDIES IN THE PROGRESSION OF FIBROSIS

In chronic hepatitis C, the transition from mild to moderate fibrosis seems to be a major prognostic step. Several consensus conferences have proposed that patients with mild fibrosis might not be treated because their prognosis is good.^1^ ^28^ ^29^ When the area of fibrosis is assessed by image analysis, the transition from moderate to severe fibrosis is associated with a marked increase in the area of fibrosis.^39^

In a previous study to identify molecular markers of prognosis in chronic hepatitis C, mRNA expression was quantified by real-time quantitative RT-PCR in a large number of selected genes in F2 (moderate fibrosis) specimens and compared with F1 (mild fibrosis) specimens.^18^ Genes involved in the physiology of fibrosis were selected (box 3). Twenty-two genes were identified that were upregulated in the F2 samples compared with the F1 samples. These upregulated genes mainly encoded genes involved in the cytoskeleton (*KRT19* and *SCG10*), growth factors/cytokines (*CXCL6*, *IL8*, *IL1A*, *IL2* and *CXCL10*), growth factor receptors (*CCR2*, *CXCR3* and *CXCR4*), in extracellular matrix production (*COL1A1*, *CHI3*, and *SPP1*), in extracellular matrix remodelling (*TIMP1*, *MMP7* and *MMP9*) and in the cell junction (*ITGA2* and *CLDN4*)(table 1).

**Table 1:** List of the top 11 genes that differ between moderate fibrosis (F2) and mild fibrosis (F1)^18^

Gene symbol	Family	Name of the encoded protein
*KRT19*	Extracellular matrix	Keratin 19
*COL1A1*	Extracellular matrix	Collagen, type I, alpha 1
*STMN2/SCG10*	Cytoskeleton	Stathmin-like 2
*CXCL6*	Cytokine	Chemokine (C-X-C motif) ligand 6
*CCR2*	Growth factor receptor	Chemokine (C-X-C motif) receptor 2
*TIMP1*	Inhibitor of matrix protease	Tissue inhibitor 1 of matrix metalloproteinase
*IL8*	Interleukin	Interleukin 8
*IL1A*	Interleukin	Interleukin 1 alpha
*ITGA2*	Cell adhesion and cell junction	Integrin alpha 2
*CLDN4*	Cell adhesion and cell junction	Claudin 4
*IL2*	Interleukin	Interleukin 2

**Box 3 Markers of matrix removal and matrix deposition**

A stellate cell is a key cell in fibrogenesis and fibrolysis. Many pathways are involved in hepatic stellate cell activation. These genes encode proteins involved in extracellular remodelling, oxidative stress, signal transduction pathways, cell cycle control, apoptosis, angiogenesis, interferon signalling, the immune response, and so forth.

**Figure:**
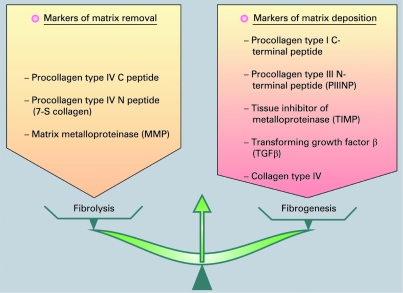

Interestingly, a recent study has demonstrated that histologically normal liver tissue obtained in two different ways (ie, percutaneous or surgical liver biopsies) has different gene expression patterns although all specimens are histologically normal.^40^ The most notable changes in gene expression occurred in the inflammatory response gene family. These results emphasise the importance of an adequate selection of histologically normal controls to prevent discordant or false results in gene expression profile analysis. Although it is difficult to state which is the best “histologically normal” control, when performing studies, histologically normal controls should always be obtained by the same technique as pathological samples. For instance, in chronic hepatitis C studies, where liver samples are obtained percutaneously, the “histologically normal” samples should also be obtained by percutaneous liver biopsy. Controls should always be clearly described. Finally, the careful selection of controls is crucial because inappropriate samples could lead to misinterpretation of results.

Finding molecular markers of the progression of fibrosis has several clinical implications. First, many of the genes found to be upregulated between mild and moderate fibrosis encode molecules secreted in the serum (cytokines). Therefore, looking for genes dysregulated in the liver can constitute a logical functional approach for the discovery of serum markers of the progression of fibrosis. For example, serum levels of cytokines have been associated with changes in the severity of chronic hepatitis C. Secondly, since a primary goal in the treatment of HCV infection is eradication of the virus and another is to stop the progression of fibrosis, molecular markers of the progression of fibrosis could help define new endpoints during antiviral treatment. Therefore, gene changes could be new markers of the progression of fibrosis during antiviral treatment. Thirdly, many of the upregulated genes identified in this gene expression study are potential molecular targets for the development of antifibrotic drugs.

## EPIGENOMICS OF HCV-INDUCED LIVER FIBROSIS

An altered pattern of epigenetic modifications is central to many common human diseases, including cancer^41^ (box 4). Although there are numerous aspects of epigenetic gene silencing, this review will focus on promoter CpG island hypermethylation, the major research area (epigenetic gene silencing) in HCV-induced liver fibrosis.

**Box 4 Epigenomics**

Human tumours undergo a massive overall loss of DNA methylation, but also acquire specific patterns of hypermethylation at certain promoters. In addition, these DNA methylation changes are linked with the presence of an aberrant pattern of histone modification. Unlike genetic alterations such as single-base mutations or deletions, epigenetic changes are potentially reversible.

DNA methylation changes are the most widely epigenetic modifications studied in humans. DNA methylation, the addition of a methyl group to the fifth carbon position of the cytosine residue, occurs in the CpG dinucleotides; 3–6% of all cytosines are methylated in normal human DNA. Potentially methylatable CpG dinucleotides are not randomly distributed in the human genome; instead, CpG-rich regions known as CpG islands, which span the 5′ end region (promoter, untranslated region and exon 1) of many genes, are usually unmethylated in normal cells. This unmethylated status corresponds with the ability of CpG island-containing genes to be transcribed in the presence of the necessary transcription factors. In cancer cells, the transcriptional silencing of tumour suppressor genes by CpG island promoter hypermethylation is key to the tumourigenic process.

DNA methylation occurs in a complex chromatin network and is influenced by the modification of histone structure. Histones are no longer considered to be simple “DNA-packaging” proteins; they are recognised as being dynamic regulators of gene activity that undergo many post-translational chemical modifications, including acetylation, methylation, phosphorylation, ubiquitylation and sumoylation. The status of acetylation and methylation of specific lysine residues contained within the tails of nucleosomal core histones is known to have a crucial role in regulating chromatin structure and gene expression, and is commonly disrupted in cancer cells.

Recent studies indicated that epigenetic alterations might initiate the expansion of premalignant cells during the early stages of tumourigenesis. During the early steps of development of the principal tumour types, such as colon, lung and prostate, but also liver tumours, a subset of these premalignant cells undergo subsequent irreversible genetic alterations that allow them to mediate tumour growth.^41^ Strategies to reverse epigenetic alterations might therefore be useful in cancer prevention and treatment.

**Figure:**
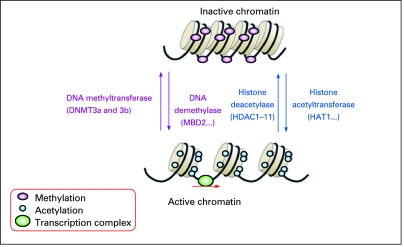

Tools for large-scale analysis of CpG island hypermethylation include array-based epigenomic analysis methods such as DHM (differential methylation hybridisation) for a genome-wide screening of CpG island hypermethylation (DNA methylome) or large-scale quantitative real-time PCR-based methylation analysis methods for more accurate and quantitative analysis of DNA methylation of specific candidate genes.^42^

There are only a few studies in the literature on array-based epigenomic analysis and the early stages of liver fibrosis associated with chronic HCV infection. Gao *et al*^43^ reported CpG island methylation at the genomic level in adjacent preneoplastic liver tissues (chronic hepatitis and liver cirrhosis) of 38 hepatocellular carcinoma (HCC) patients with HCV infection (n = 15), HBV infection (n = 11), both (n = 1) or without viral infection (n = 11). These authors identified a large number of aberrant methylations involved in the transition between normal tissue and adjacent tissue with not further increase in cancer (ie, *MMP14*, *miR-219*, *CIDEA*, etc.) or in the transition between tissues adjacent to HCC (ie, *RASFF1A*, *TBX4*, *CA10*, *PTPRT*, etc.).

Several studies using quantitative real-time PCR-based methylation analysis for DNA methylation of specific candidate genes suggest that DNA methylation is an early event preceding cirrhosis.^44^^–^49 Other studies suggest that some of the aberrant methylation in early-stage hepatitis is part of the normal ageing process, chronic inflammation, iron overload and persistent viral infections (in particular HCV). Nishida *et al* report that aberrant methylation of a limited number of loci is common in the normal ageing liver and that these epigenetic alterations gradually progress and expand to a larger panel of methylation markers in HCC.^44^ Moreover, they have observed that persistent viral infection, in particular HCV, accelerates age-related methylation in the liver, suggesting that aberrant methylation may play an important role in the genesis of HCC. Exposure to the major epimutagen HCV induces methylation and causes disruption of numerous genes and pathways during fibrinogenesis and hepatocarcinogenesis. The pathways showing epigenetic alterations in various stages of liver fibrosis (up to cirrhosis) include: cell proliferation (*p16*, *RIZ1/PRDM2*, *HIC-1*, etc.), the Wnt/β-catenin pathway (*APC*, *CDH1, SFRP1-4*, etc.), the Ras pathway (*SPROUTY* and *RASSF1A*), the Jak–STAT pathway (*SOCS1*, *SOCS3*), the TGFα pathway (*RUNX3*) and metabolism of xenobiotics (*GSTP1*, etc.).

### The future of HCV-induced liver fibrosis epigenomics

Although interesting biological insights and promising tools are being developed, HCV-induced liver fibrosis epigenomics is still in its infancy. The impact of the powerful epimutagen HCV on the progression of aberrant methylation must be elucidated for the different steps of liver fibrosis. For this, comprehensive epigenomic profiling must be performed at every step of liver fibrogenesis as well as hepatocarcinogenesis. Gene silencing can be viewed as an “epigenetic gatekeeper” step that creates a milieu that facilitates the selection of mutations in oncogenes and tumour suppressor genes promoting tumour progression.

Understanding the molecular events that initiate and maintain epigenetic gene silencing in the early stages of HCV-associated fibrosis could help in the development of strategies to reverse the silencing process for the prevention and treatment of cirrhosis and HCC.

## MIRNAOME AND LIVER FIBROSIS ASSOCIATED WITH CHRONIC HCV INFECTION

Recent evidence has shown that miRNA mutations or misexpression are associated with various human cancers and that miRNAs can function as tumour suppressors and oncogenes.^50^ Oligonucleotide miRNA microarray analysis is the most commonly used high-throughput technique to assess miRNA expression profiling (miRNAome) for a large series of sample. The expression of a selection of a smaller number of miRNAs can be determined by quantitative real-time RT-PCR.

There are a few pilot studies in the literature on miRNA expression profiling in hepatocellular tumours, but few or none concerning HCV-induced liver fibrosis.

The studies in HCC tissues or experimental models of HCC have all shown that specific miRNAs are aberrantly expressed in malignant HCC cells or tissues compared with non-malignant tissues.^51^^–^53 Thus these miRNAs may provide insight into cellular processes involved in carcinogenesis or be markers of malignancy. The first miRNAs shown to be increased (miR-21 and miR-224) or decreased (miR-199a and miR-122a) in HCC must be confirmed in a large series of tumours. Several of these miRNAs have been shown to target specific genes directly in liver tissue—that is, miR-21 regulates PTEN,^52^ miR-122a regulates cyclin G1,^53^ while miR-221 controls p57/CDKN1C and p27/CDKN1B.^54^ It is also important to note that some miRNA genes (such as miR-1) undergo methylation-mediated regulation in HCC cell lines,^55^ suggesting a strong link between the DNA methylome and the miRNAome. Interestingly, the miRNA expression profiles differ between malignant hepatocytes, malignant cholangiocytes^56^ and benign liver cancer,^57^ suggesting that miRNA profiling could be used as molecular diagnostic markers in liver disease.

Although there are few studies in the literature on miRNA genes and HCV-induced liver fibrosis, we hypothesise that miRNAs play a critical role in HCV infection, in the progression of fibrosis and in the prediction of treatment response. Jiang *et al* suggest that important changes in miRNA expression occur during the development of chronic viral hepatitis and cirrhosis.^58^

## RESPONSE TO TREATMENT

### Existing predictive factors of response to treatment

A sustained virological response (SVR) rate of ∼55% is obtained with the combination of pegylated IFNs (PEG-IFNs) and ribavirin.^59^^–^62 Long-term follow-up studies showed that an SVR is generally associated with clinical and histological improvement and eradication of HCV infection in most patients.^63^

Since a significant number of patients will fail to respond to treatment, or have a virological relapse or significant side effects so that treatment must be stopped, it is important to identify non-responding patients as early as possible and ideally at baseline (before treatment) both for patient welfare and for cost-effectiveness. The probability of an SVR essentially depends on genotype. Younger age, female gender, mild fibrosis and low viral load are also associated with a better response rate, but to a lesser extent. In patients with HCV genotypes 2 or 3, the SVR rates reach 80%; in genotype 1 patients the SVR rate is 50%.

An early virological response is the best predictive factor of an SVR.^60^ A reduction in HCV RNA serum levels by <2 log10 copies/ml after the first 12 weeks of treatment compared with baseline is clearly associated with almost no chance of an SVR (negative predictive value, 97–100%). A rapid virological response (undetectable HCV RNA at week 4) seems to be the best predictor of treatment outcome in patients with chronic hepatitis C.^64^

### Large-scale and genome-wide association studies, and response to treatment

Hwang and colleagues have used this method to look at the genetic differences that were associated with IFN/ribavirin responses in 317 Taiwanese patients with chronic hepatitis C.^65^ The panel of 26 SNPs in seven genes associated with treatment responsiveness (*ADAR*, *CASP5*, *FGF1*, *ICSBP1*, *IFI44*, *TAP2* and *TGFBRAP1*) was used to construct a model by multiple logistic regression. The sensitivity and the specificity of the model were 68.2% and 60.7%, respectively. Nonetheless, there are several weaknesses in the logistic regression approach. First, it cannot detect complex gene–gene interactions since, like other traditional regression methods, it relies on the basic assumption of linear combinations only. Secondly, the rapid increase in the availability of large numbers of genetic markers increases the number of potential predictors considerably, which, when combined with the significantly smaller number of observations, creates a statistical problem called the ‘curse of dimensionality’.^66^ Therefore, higher order computational methods are needed to select a small group of predictors and/or interactions between predictors with a significant effect on the trait of interest from the numerous genetic and environmental predictors

Several “machine learning” approaches that have recently been applied to investigate potential gene–gene and gene–environment interactions in IFN treatment or in the risk of developing cirrhosis have been highly successful for modelling the relationship between combinations of polymorphisms and clinical endpoints.^36^ ^67^^–^69

Lin *et al* used an artificial neural network (ANN) to address gene–gene and gene–environment interactions in antiviral treatment response for 523 patients with chronic hepatitis C who had received IFN and ribavirin combination treatment, including 350 sustained responders and 173 non-responders.^68^ They focused on candidate genes involved in pathways related to IFN signalling and immunomodulation. A total of 20 SNPs were selected from six candidate genes (*ADAR*, *CASP5*, *ICSBP1*, *IFI44*, *PIK3CG* and *TAP2*). A feedforward neural network was used to model the responsiveness of IFN, and the back-propagation algorithm was used for the learning scheme. Inputs were genetic and clinical factors including SNP markers, viral genotype, viral load, age and gender. Outputs were the IFN-responding status. The prediction accuracy of each model was estimated using a fivefold cross-validation procedure, and a permutation test was applied to measure the statistical significance of an association between predictors and drug response. All possible combinations of *N* factors were evaluated sequentially and the *N*-factor model displaying the highest prediction accuracy was retained. *IFI44* was found in the significant two-locus, three-locus and four-locus gene–gene effect models as well as in the significant two-factor and three-factor gene–environment effect models. Furthermore, viral genotype remained in the best two-factor, three-factor and four-factor gene–environment models. Thus, these results strongly support the hypothesis that viral genotype and *IFI44*, a member of the family of IFN-inducible genes, play a role in the pharmacogenomics of IFN treatment. In addition, this approach identified a panel of 10 factors that may be more significant than the others for further investigation. Hence, theses results suggest that an ANN-based approach may be useful to analyse the complex non-linear relationship between genetic and clinical factors and the responsiveness of IFN. Similarly, a support vector machine (SVM) algorithm was applied to build a tool to predict responsiveness to IFNα–ribavirin combination treatment using host polymorphisms and viral genotype.^69^ These authors concluded that the SVM algorithm is a promising method to evaluate the complex non-linear relationship between factors and successful treatment to predict response to IFNα.

Another study genotyped eight SNPs spanning the entire IFNγ gene in two cohorts and assessed the association between those polymorphisms and treatment response or spontaneous viral clearance.^70^ The first cohort was composed of 284 patients with chronic hepatitis C who had received IFNα-based treatment and the second comprised 251 intravenous drug users who had either spontaneously cleared HCV or become chronically infected. An SNP variant located in the proximal IFNγ promoter region next to the binding motif of heat shock transcription factor (HSF), –764G, was significantly associated with SVR. The association was independently significant in multiple logistic regression (p = 0.04) along with race, viral load and genotype. This variant was also significantly associated with spontaneous recovery in the second cohort. Functional analyses show that the G allele confers a two- to threefold higher promoter activity and stronger binding affinity for HSF1 than the C allele. This study suggests that the IFNγ promoter SNP –764G/C is functionally important in determining viral clearance and treatment response in HCV-infected patients.

### Liver molecular signature of response

Liver gene expression profiling has recently been applied to chronic hepatitis C to determine response to treatment. Knowledge of the antiviral mechanisms of IFN is crucial for the discovery of new treatment response markers (fig 1). In a recent study, expression profiling was performed on percutaneous needle liver biopsy specimens taken before treatment.^71^ Gene expression levels were compared in 15 non-responder, 16 responder and 20 normal liver biopsy specimens. The authors identified 18 genes whose expression differed significantly between all responders and all non-responders. Many of these 18 genes are IFN sensitive, and two (ISG15/USP18) are linked in a novel IFN regulatory pathway, suggesting a possible rationale for treatment resistance. In another study, liver tissue samples were analysed by microarray prior to treatment by IFN or IFN/ribavirin.^72^ In the IFN group, the differentially expressed genes were mainly IFN-, lipid metabolism-, complement- and oxidoreductase-related genes. In the IFN/ribavirin combination group, a different set of genes was identified with cyclophilin A and multidrug resistance protein.

In a recent study, a selection of genes associated with liver gene expression dysregulation during HCV infection was studied by large-scale RT-PCR assay according to response to treatment.^73^ Supervised class prediction analysis identified a two-gene (*IFI27* and *CXCL9*) signature, which accurately predicted treatment response in 79.3% (23/29) of patients from the validation set (Group B), with a predictive accuracy of 100% (9/9) and of 70% (14/20) in non-responders and sustained virological responders, respectively. The expression profiles of responder–relapsers did not differ significantly from those of non-responders and sustained virological responders, and 73% (8/11) of them were predicted as sustained virological responders with the two-gene classifier. In conclusion, non-responders and sustained virological responders have different gene expression profiles prior to treatment. The most notable changes in gene expression were mainly observed in the IFN-stimulated genes (table 2). We were able to predict treatment response with a two-gene signature (*IFI27* and *CXCL9*) in two independent groups of patients (training and validation set). Most relapsers clustered with sustained virological responders. Interestingly, the baseline liver levels of expression of IFN-stimulated genes were higher in non-responderss than in sustained virological responders. The failure to respond to exogenous PEG-IFN in non-responders could indicate a blunted response to IFN. This suggests that IFN-stimulated genes are already maximally induced in non-responderss. Genes included in the signature encode molecules secreted in the serum and provide a logical functional approach for the development of serum markers to predict response to treatment.

**Table 2:** List of the genes that differ between non-responders and sustained virological responders (training set)^73^

Gene symbol	Family	Name of the encoded protein	NR/SVR
*IFI-6-16*	IFN-inducible protein	IFNα-inducible protein 3	3.5
*IFI27*	IFN-inducible protein	IFNα-inducible protein 27	4.2
*ISG15*	IFN-inducible protein	IFNα-inducible protein 2	3.7
*MX1*	IFN-inducible protein	Activating transcription factor 6	2.7
*HERC5*	IFN-inducible protein	Hect domain and RLD 5	2.2
*TGFB2*	Growth factor	Transforming growth factor β2	2.7
*OAS2*	IFN-inducible protein	29-59-Oligoadenylate synthetase 2	1.8
*VEGFD*	Angiogenesis	Vascular endothelial growth factor D	2.4
*IL8*	Interleukin	Interleukin 8	3.2
*IFIT1*	IFN-inducible protein	IFN-induced protein with tetratricopeptide repeats 1	55.3

Gene expression ratios were compared among non-responder and sustained virological responder liver gene expression values.

IFN, interferon.

One study analysed gene expression patterns in peripheral blood mononuclear cells during IFN treatment and confirmed upregulation of genes thought to be IFN-stimulated genes or involved in antigen processing and presentation.^74^ However, studies assessing gene expression in peripheral blood mononuclear cells in chronic hepatitis C are lacking.

Interestingly, data in HCV-infected chimpanzees indicate a predominantly defective hepatic response to IFN, which is probably mediated through the activation of SOCS3 and may explain the lack of response to IFN-based treatment in many HCV patients.^75^ Furthermore, an association study tested three SNPs of SOCS3 in 162 non-responders and 184 sustained responders (SOCS3 –8464 A/C (rs12952093), –4874 A/G (rs4969170) and 1383 A/G, (rs4969168)).^76^ SOCS3 basal expression levels were significantly increased in two independent sets of non-responder groups. The SOCS3 –4874 AA genotype was strongly associated with antiviral treatment failure, and AA genotype carriers had significantly higher SOCS3 mRNA and protein levels

The diversity of microarray platforms utilised for gene expression analysis and the variability in microarray data emphasise the need for quality assurance. High quality RNA samples are essential for gene expression analysis, and the quality of each RNA preparation must be rigorously assessed. Careful measures must be taken during all steps of the RNA extraction to prevent the RNA from degrading. Furthermore, improved analytical procedures and the use of large numbers of patients are needed for validation. Gene signatures will probably be used in the future for optimised and tailored treatment.

Many of the genes found to be upregulated between non-responders and responders encode molecules secreted in the serum (cytokines) and are a logical functional approach for the development of serum markers as predictors of response to treatment. For instance, one study examined the levels of chemokines that bind to CXC chemokine receptor 3 (CXCR3) to determine whether they play a role in the failure of the immune system to clear HCV infection.^77^ Levels of CXCL10 and CXCL9 decreased during successful antiviral treatment; CXCL11 did not decrease significantly during treatment or in the first 6 months after treatment. Baseline levels of CXCL10 were highest in HCV patients who did not respond to treatment. These results suggest that plasma concentrations of immunoreactive CXCL10 may be a predictor of response to PEG-IFN with or without ribavirin. It should be noted that we identified CXCL9 in our two-gene signature, and that CXCL9, CXCL10 and CXCL11 have the same specific receptor CXCR3.^73^

## THE PROTEOME IN DIAGNOSTIC AND TREATMENT

Characterisation of the liver and serum proteomes is the next step in the investigation of patients with liver diseases since protein signatures are a potentially powerful tool in the diagnosis and prognosis of patients with HCV infection. Although there are several studies describing global gene expression changes associated with HCV infection, changes in the proteome have not been extensively characterised. Proteomics uses a combination of sophisticated techniques including 2D gel electrophoresis, image analysis, mass spectrometry, amino acid sequencing and bioinformatics.^78^ A major hurdle when studying proteins is the potentially enormous number of proteins present in a biological sample. This diversity is due to the many post-transcriptional and post-translational modifications that proteins can display. Another difficulty is the wide range of concentrations of highly represented proteins and the low concentration of potential biomarkers. Without specific protein amplification techniques such as PCR for nucleic acids, the identification of biomarkers requires technologies which have not yet been fully validated. However, there are several good reasons to focus on protein analysis: (1) the level of mRNA expression does not often parallel the amount of protein; (2) study of the genome does not address dynamic cell processes; and (3) recent improvements in proteomic technologies, such as proteomic profiling technology, provide global visualisation of the proteome by a high-throughput method and lead to the identification of isolated or clustered peaks associated with a disease in a complex biological sample.

There are very few studies in the literature addressing proteomic changes in hepatitis C. Jacobs *et al* provide a global proteome analysis of changes induced by HCV infection by multidimensional liquid chromatographic separation coupled with mass spectrometry in the full-length HCV replicon model.^79^ Several proteins involved in lipid biosynthesis were found to be upregulated, while proteins involved in fatty acid oxidation were downregulated. Some of these proteins were also found to be deregulated in liver biopsies of patients with chronic HCV infection. These data support the suggested relationship between HCV and lipid metabolism, although the physiopathological relevance of these data needs to be investigated further.

Liver fibrosis and cirrhosis are the consequences of chronic HCV infection. They are characterised by marked modifications of proteins including synthesis of extracellular matrix proteins, suggesting that proteomics might provide new insights for diagnosis of fibrosis or cirrhosis. Several studies have been performed to characterise protein changes in tissues in experimental models of liver fibrosis. For instance, several proteins that are significantly deregulated during liver fibrosis have been identified by 2D gel electrophoresis and mass spectrometry.^80^ Using similar experimental animal models and culture of hepatic stellate cells (HSCs), Kristensen *et al* compared proteomic dynamics in in vitro and in vivo processes of HSC activation.^81^ The expression of 43 proteins was shown to be altered by 2D gel electrophoresis, when the cells were activated in vivo and/or in vitro. Among them, 27 displayed similar changes in vivo and in vitro, including two members of the S100 protein family (calcyclin and calgizzarin) and galectin-1 (a galactosidase-binding lectin) involved in growth regulation and neoplastic transformation.

To date, few studies have focused on serum protein changes during the development of liver fibrosis. In thioacetamide-induced liver fibrosis, Xu *et al* showed that expression of 59 protein spots significantly changed upon thioacetamide administration and that a protein of 3.495 kDa, sharing homology with a histidine-rich glycoprotein, was consistently decreased in sera of cirrhotic rats.^82^ In addition, in one study, Poon *et al* tried to define serum protein signatures associated with various degrees of fibrosis and develop a proteomic fingerprinting model for predicting fibrosis and cirrhosis in patients with chronic hepatitis B infection.^83^ For this purpose, they developped ANN models to generate an ANN fibrosis index based on the proteomic data obtained by ProteinChip Array with laser desorption-ionisation time of flight mass spectrometry (SELDI-TOF-MS ProteinChip technology). SELDI-TOF provides rapid protein profiles and comparative analyses according to patient phenotype.^83^ This approach takes advantage of an initial fractionation step of the proteome according to fixation on different affinity surfaces, followed by desorption and time-of-flight analysis of retained proteins. This technique has several advantages such as being easy to use and high throughput, making it compatible with clinical proteomics. Thirty protein changes were defined that were significantly associated with the extent of liver fibrosis.^83^ Interestingly, some of these protein peaks were correlated with biological data exploring liver function, such as albumin. Finally, this study showed that the ANN fibrosis index derived from the serum proteomic fingerprint predicts patients with significant fibrosis and cirrhosis. Using the same approach, we demonstrated that antiviral treatment induced chronological changes in the serum proteome and that these variations were dependent upon virological response to treatment.^84^ Moreover, serum proteome analysis in naive patients predicted SVR to PEG-IFN plus ribavirin in most of our patients. Molecular identification of the peaks isolated by the SELDI-TOF approach would be of major interest for developing serum tests for patient care. Although these global approaches are quite effective and are well adapted to clinical proteomics. they have several limitations including the difficult molecular identification of protein peaks and the necessity for further analysis based on purification, peptide fingerprinting and MS/MS sequencing. This is the major limitation of global proteomics analyses at present.

## CONCLUSION

In addition to viral and environmental behavioural factors, host genetic diversity is believed to play a role in each step of the different clinical outcomes in HCV infection (clearance of acute infection, progression of fibrosis and treatment outcome). The sequencing of the human genome, together with the development of high-throughput technologies that identify gene function, have provided unique opportunities to develop profiles to distinguish, identify and classify discrete subsets of the disease, predict disease outcome or predict response to treatment. Multiplexed protein measurement (such as flow cytometric microbead assays using Luminex technology) is a rapidly advancing field that has the broadest potential technology to transform clinical diagnostic in the next 10 years.

In the near future, further studies must meet several challenges: first, studies with large prospective cohorts with well phenotyped patients are needed. For instance, in the studies evaluating the severity of the disease, the stage of fibrosis (scoring systems for histological fibrosis) must be carefully determined, taking into account the duration of HCV infection. For response to treatment, studies must use the same definition of SVR and the same treatment regimen. Statistical analysis must take into account all factors associated with fibrosis progression or response to treatment. Secondly, technologies must be improved for routine use. For instance, the diversity of microarray platforms used for gene expression analysis and the variability in microarray data emphasise the need for quality control. Careful measures must be taken during all steps of RNA extraction to prevent the RNA from degrading. Furthermore, analytical procedures must be improved and large numbers of patients must be studied for validation. Gene signatures will probably be used in the future for personalised medicine (optimised and tailored treatment).
